# Revealing the Origin and Nature of the Buried Metal‐Substrate Interface Layer in Ta/Sapphire Superconducting Films

**DOI:** 10.1002/advs.202413058

**Published:** 2025-02-19

**Authors:** Aswin k. Anbalagan, Rebecca Cummings, Chenyu Zhou, Junsik Mun, Vesna Stanic, Jean Jordan‐Sweet, Juntao Yao, Kim Kisslinger, Conan Weiland, Dmytro Nykypanchuk, Steven L. Hulbert, Qiang Li, Yimei Zhu, Mingzhao Liu, Peter V. Sushko, Andrew L. Walter, Andi M. Barbour

**Affiliations:** ^1^ National Synchrotron Light Source II Brookhaven National Laboratory Upton New York 11973 USA; ^2^ The Condensed Matter Physics and Materials Science Department Brookhaven National Laboratory Upton New York 11973 USA; ^3^ Center for Functional Nanomaterials Brookhaven National Laboratory Upton New York 11973 USA; ^4^ IBM T. J. Watson Research Center 1101 Kitchawan Road Yorktown Heights New York 10598 USA; ^5^ Department of Materials Science and Chemical Engineering Stony Brook University Stony Brook New York 11794 USA; ^6^ Material Measurement Laboratory National Institute of Standard and Technology Gaithersburg Maryland 20899 USA; ^7^ Department of Physics and Astronomy Stony Brook University Stony Brook New York 11794 USA; ^8^ Physical and Computational Sciences Directorate Pacific Northwest National Laboratory Richland Washington 99354 USA

**Keywords:** density functional theory modeling, HAADF‐STEM, superconducting films, synchrotron X‐ray reflectivity, tantalum

## Abstract

Despite constituting a smaller fraction of the qubit's electromagnetic mode, surfaces and interfaces can exert significant influence as sources of high‐loss tangents, which brings forward the need to reveal properties of these extended defects and identify routes to their control. Here, we examine the structure and composition of the metal‐substrate interfacial layer that exists in Ta/sapphire‐based superconducting films. Synchrotron‐based X‐ray reflectivity measurements of Ta films, commonly used in these qubits, reveal an unexplored interface layer at the metal‐substrate interface. Scanning transmission electron microscopy and core‐level electron energy loss spectroscopy identified an intermixing layer (≈0.65 ± 0.05 nm) at the metal‐substrate interface containing Al, O, and Ta atoms. Density functional theory modeling reveals that the structure and properties of the Ta/sapphire heterojunctions are determined by the oxygen content on the sapphire surface prior to Ta deposition for two atomic terminations of sapphire. Using a multimodal approach, we gained deeper insights into the interface layer between the metal and substrate, which suggests that the orientation of deposited Ta films depend on the surface termination of sapphire. The observed elemental intermixing at the metal‐substrate interface influences the thermodynamic stability and electronic behavior of the film, which may also affect qubit performance.

## Graphical abstract

The origin and nature of metal‐substrate interface layer in Ta/sapphire‐based superconducting films is investigated using synchrotron X‐ray reflectivity and scanning transmission electron microscopy. An intermixing layer, ≈0.65 nm thick containing Al, O, and Ta atoms, is identified at the interface. Density functional theory modeling revealed that Al‐rich surfaces promote significant Ta‐Al intermixing matching experimental measurements.

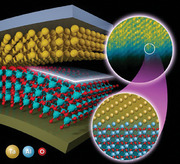

## 1 Introduction

In recent years, there has been significant progress in the development of platforms aimed at revolutionizing quantum computing hardware^[^ ^1^ ^]^ These advancements have the potential to address issues in fields like materials science and cryptography that would traditionally take decades to solve using classical computers. A breakthrough in quantum computing hardware has been the discovery of superconducting quantum circuits (SQC).^[^ ^2^ ^]^ SQC offer a promising approach for quantum computing due to their low error rates and scalability. However, the practical implementation of superconducting qubits in a quantum processor is hindered by their limited coherence lifetime (*T*_1_), which, in turn, is thought to be primarily limited by microwave dielectric losses^[^ ^3^ ^]^ Various research groups are actively working to improve the *T*_1_ lifetime through two main approaches: understanding the microscopic mechanisms to better control performance and exploring new material choices^[^ ^4^ ^]^

Among the candidate materials for the microwave resonator, such as metal nitrides^[^ ^5^ ^]^ aluminum (Al),^[^ ^6^, ^7^ ^]^ niobium (Nb)^[^ ^8^ ^]^ and tantalum (Ta),^[^ ^9^, ^10^ ^]^ a major improvement in the state of art was realized with Ta qubits, which were the first to achieve T1 over 0.3 ms^[^ ^9^ ^]^ Additionally, a recent study by Tuokkola et al., demonstrated that Al based microwave resonators can achieve T_1_ lifetimes of ≈1 ms^[^ ^11^ ^]^ While Ta based superconducting qubits show higher coherence lifetimes than many other qubit materials, Ta still faces challenges, such as surface oxide formation as soon as the films are removed from the ultra‐high vacuum environment. This surface oxide layer is usually disordered, leading to two‐level system (TLS) loss in superconducting transmon qubits, which in turn results in a dielectric loss in the qubit, thereby impacting the *T*_1_.^[^ ^12^, ^13^ ^]^

Recently various research groups (including ours) focused on a more detailed analysis of the Ta interfaces between the air and metal layer. This analysis necessitated the use of advanced characterization tools including synchrotron‐based variable energy X‐ray photoemission spectroscopy (VEXPS) and transmission electron microscopy (TEM) measurements to understand the growth mechanisms of this Ta_2_O_5_ layer and provide insights on the structure and composition of this Ta_2_O_5_/Ta (oxide‐metal) interface.^[^ ^8^, ^14^, ^15^ ^]^ These measurements provided further evidence that the oxide‐metal interface in Ta transmon qubits is chemically less complex than those observed in Nb transmon qubits^[^ ^8^ ^]^ One avenue receiving much attention is suppressing the formation of the surface oxide layer. An effective strategy for suppressing the surface oxide formation is the surface encapsulation of the Ta or Nb films before exposing them to the ambient air environment, with transition metals.^[^ ^16^, ^17^ ^]^ However, all these works discuss the oxide formation at the metal‐air (M‐A) interface and its impact on the *T*_1_ of a superconducting transmon qubit.

There are also other interfaces, such as the substrate‐air (S‐A) and the metal‐substrate (M‐S) interfaces, which also likely give rise to TLS.^[^ ^18^, ^19^, ^20^ ^]^ Wenner et al. used COMSOL simulations to demonstrate that surface losses in superconducting coplanar waveguide resonators are dominated by the substrate‐vacuum and metal‐substrate interfaces, which are 100 times more lossy than the metal‐vacuum interface.^[^ ^21^, ^22^ ^]^ Similarly, Wang et al. showed that dielectric dissipation arising from material interfaces is a major limiting factor for the T_1_ of transoms in 3D circuit quantum electrodynamics architectures^[^ ^23^ ^]^ More recently, Oh et al. found that an amorphous Nb_x_Si_y_ interface layer between Nb and the Si substrate significantly contributed to TLS losses in Nb/Si based coplanar waveguide resonators^[^ ^24^ ^]^ However, to the authors’ knowledge, there has been no experimental or theoretical study reported on the M‐S interface in the case of Ta/sapphire based superconducting qubits. Understanding the linkages between the synthesis/processing conditions, the resulting structure, fundamental properties, and the performance of these qubits is necessary to enable significant advancements in quantum computing technology.

In this work, we investigated the extent of structural and compositional inhomogeneity at the M‐S interface of a superconducting Ta film (30 nm) on c‐plane sapphire. The interfacial chemical states and roughness of the Ta film were examined using variable‐energy X‐ray photoelectron spectroscopy (VEXPS) and X‐ray reflectivity (XRR). The phase purity of the Ta film and the epitaxial relation of the film were evaluated by X‐ray diffraction (XRD) and transmission electron microscopy (TEM) techniques, respectively. Chemical profile analysis via high‐angle annular dark‐field scanning transmission electron microscopy (HAADF‐STEM) and electron energy loss spectroscopy (EELS) was performed to obtain depth profile information across the metal‐substrate interface. Density function theory (DFT) modeling was used to determine the origin of the M‐S interface structural ordering using different sapphire termination models. Combining insights obtained from XRR and STEM experiments and DFT modeling, we reveal the presence of an unexplored layer at the M‐S interface, which is formed by the intermixing of Ta, Al, and O atoms from the metal and substrate layers. These findings offer valuable insights into controlling the structure and composition of the substrate‐metal interface, thus paving the possible way to longer qubit coherence times.

## 2 Results and Discussion

A comprehensive description of the Ta thin film deposition process, the characterization methods employed in this study, and the DFT modeling approach can be found in the supporting information. XRR measurements were performed on Ta film using lab‐based and synchrotron‐based X‐ray sources to determine the thickness, electron density, and roughness (Figure S1, Supporting Information). The XRR of Ta films demonstrates a sharper interface in the Ta film, as indicated by the oscillation fringes up to a *Q_z_* value of 1.4 Å^−1^ (where *Q_z_* = 4πsin*θ*/*λ*, *θ*, and *λ* represent the Bragg angle and incident X‐ray wavelength, respectively) in the case of synchrotron‐based measurements compared to lab‐based measurements. The poorer statistics at higher values of *Q_z_* in lab‐based instruments (Figure S1a, Supporting Information) are due to the lower X‐ray beam flux and hence lower detector/energy resolution. In Figure S1b (Supporting Information), the samples were measured at different φ (azimuthal) angles to determine any influence on the roughness of the Ta film due to possible surface terracing resulting from a surface miscut. Where φ represents the sample stage's rotation around the sample's normal axis. Upon comparison of the two phi angles, we find that φ = 0° has the slightly sharper interfaces.

**Figure 1:**
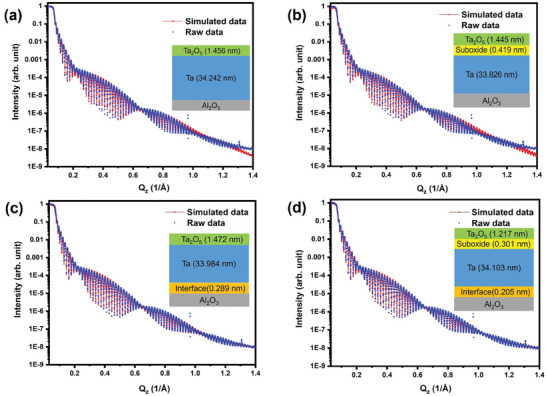
Fittings of the synchrotron X‐ray reflectivity measurements for the BCC Ta film on sapphire substrate according to different layer models. a) Model 1 neglecting transitional (buffer) layers, b) Model 2 with an interfacial buffer layer between the Ta_2_O_5_ and metal layers, c) Model 3 accounting for an interface layer between the substrate and metal layers, and d) Model 4 with interface layers between both the Ta_2_O_5_/metal and substrate/metal layers. The insets show the corresponding fitted thickness values obtained.

The synchrotron‐based XRR measurements are fitted with different models using the genX software^[^ ^25^ ^]^ (**Figure** **1**). Figure 1a depicts a simple fitting model (Model 1), consisting of a slab of sapphire with a layer of Ta and a native Ta oxide (Ta_2_O_5_) above it. However, the fitting is poor at higher Q_z_ values. In Figure 1b, an interface layer of mixed suboxide was introduced at the M‐A interface (i.e., Ta_2_O_5_/sub‐oxide interface/Ta metal) (Model 2). The parameters used for this suboxide interface model are based on the findings from our previous work^[^ ^13^ ^]^ There have been slight changes in the fitting parameters from this fitting model, but the fitting remained poor at higher Q_z_ values. Figure 1c shows the XRR fitting results after incorporating an interface layer at the M‐S region, which we refer to as the metal‐interface‐substrate (M‐I‐S) layer (Model 3). This model is more realistic than models 1 and 2, as the fitting improved at higher Q values. We also considered another fitting model (Figure 1d), in which we combined Models 2 and 3, incorporating an interface suboxide layer at the M‐A interface and an interface layer at the M‐S interface (Model 4). This model provided similarly improved results compared to the model in Figure 1c. These findings provide the evidence of an existence of a sharp and thin layer between the Ta metal and substrate layer (i.e., the M‐I‐S layer) that has a thickness of 0.205 ± 0.047 nm. The interfacial roughness of this layer is 0.145 ± 0.013 nm, which corresponds to the interface between the blue and orange‐colored slabs as shown in Figure 1d. The value of 0.051 ± 0.015 nm represents the roughness at the interface‐substrate layer, corresponding to the interface between the orange and grey‐colored slabs. Here the presented error bars are generated by the genX software and represent a 5% increase to the fitting figure of merit (FOM). Figure S2 (Supporting Information) shows the XRR fitting results along with the residuals for Model 4. The fitting results for the data in Figure 1c,d are summarized in **Table** **1**. Additionally, these measurements suggest the importance of obtaining a better S/N ratio at higher Q_z_ values, especially Q_z_ >1.2 Å^−1^ in this case to observe this metal‐interface‐substrate (M‐I‐S) layer.

**Table 1:** Comparison of the Ta/sapphire heterojunction characteristics obtained by fitting XRR measurements using Models 3 and 4.

Parameters	Model 3 (units in nm)	Model 4 (units in nm)
Thickness of Ta_2_O_5_	1.472 ± 0.112	1.217 ± 0.122
Thickness of top suboxide layer	NA	0.301 ± 0.135
Thickness of Ta	33.984 ± 0.047	34.103 ± 0.020
Thickness of bottom interface layer (M‐I‐S)	0.289 ± 0.022	0.205 ± 0.047
Roughness of Ta_2_O_5_	0.235 ± 0.033	0.234 ± 0.042
Roughness at the top suboxide layer	NA	0.204 ± 0.137
Roughness of Ta	0.227 ± 0.038	0.195 ± 0.049
Roughness at the Ta metal – interface layer	0.067 ± 0.030	0.145 ± 0.013
Roughness at the interface‐substrate layer	0.109 ± 0.012	0.051 ± 0.015

Similar findings have been reported earlier in the case of Nb films (40 nm), where researchers demonstrated that their XRR measurements were best modeled by the addition of thin interface layers at the Nb‐sapphire interface.^[^ ^26^, ^27^ ^]^ In a recent work, Satchell et al.^[^ ^28^ ^]^ also confirmed that an additional interface layer is required to fully model the XRR measurements on a Nb (65 nm films)/sapphire substrate. The origin of this interface layer may correspond to either alloying or chemical reactions between them, leading to a possible formation of an NbAl layer. These previous reports on Nb films suggest that the thin, unexplored interface layer in Ta film could be due to the possible intermixing of Ta and sapphire atoms.

**Figure 2:**
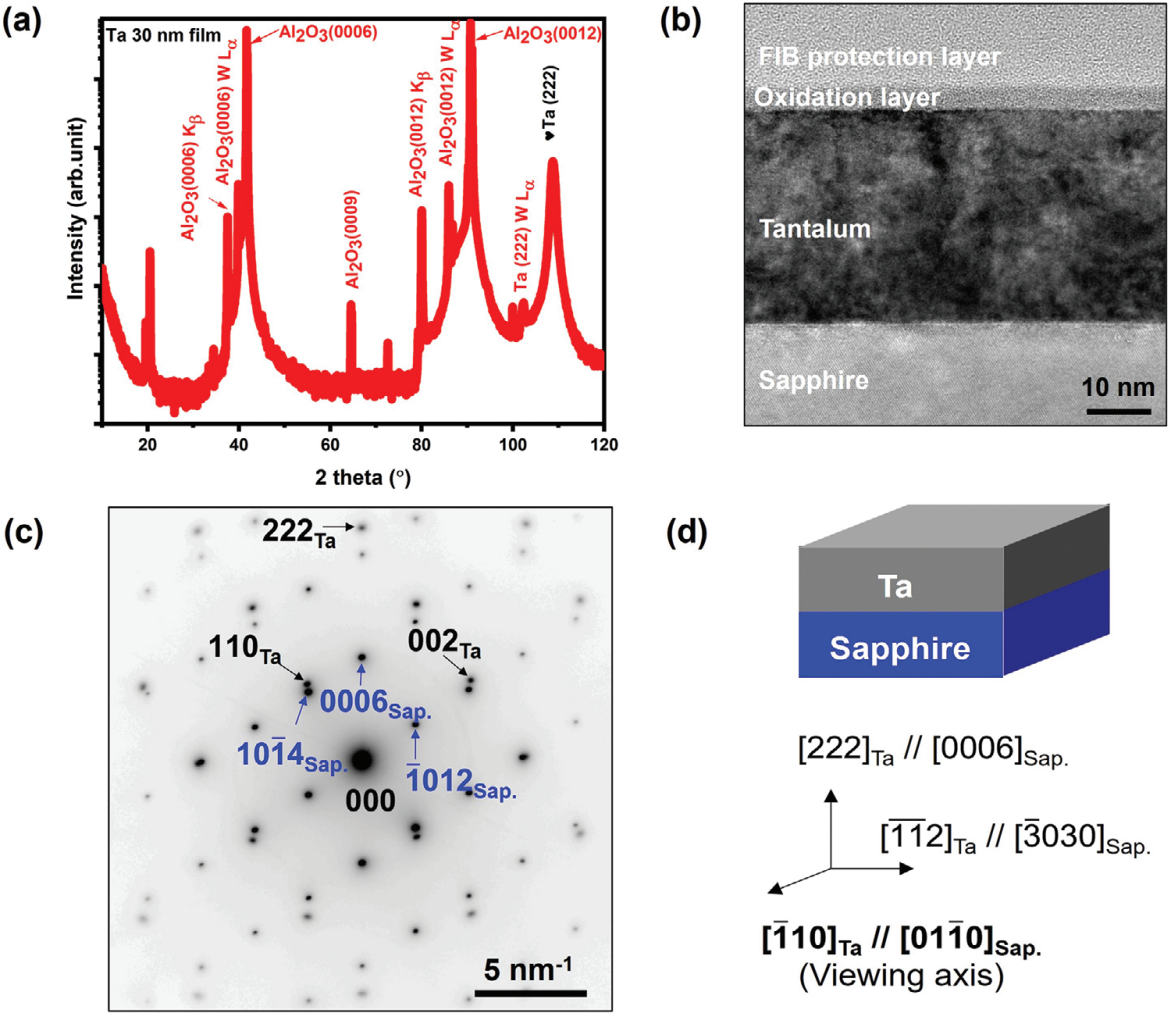
Analysis of the Ta film properties. a) XRD pattern of an *α*‐Ta film on C‐plane sapphire, b) cross‐section TEM image of the Ta film, c) the SAED pattern illustrating the epitaxial relationship between the Ta film and the substrate, and d) the schematic representation of a 30 nm thick *α*‐Ta film on c‐plane sapphire substrate.

XRD measurements were performed to determine the crystal structure of the sputtered Ta thin film on c‐plane sapphire (0001). The out‐of‐plane XRD measurements (θ‐2θ scan) shown in **Figure** **2**a, confirmed that the Ta film exists in the pure BCC structure with a preferred orientation along the (222) direction, which is consistent with the orientation previously reported by Yao et al^[^ ^29^ ^]^ Figure 2b shows the cross‐section TEM image of the Ta film with a thickness of ≈34.0 ± 0.5 nm, consistent with the XRR fitting results. Additionally, a thin layer of ≈4.0 ± 0.2 nm of amorphous native surface oxide exists on top of the Ta film. Here the measurement represents the average and standard deviation of ten unique measurements. The selected area electron diffraction (SAED) pattern (Figure 2c) illustrates that the TEM sample was measured along the [1̅10] direction of Ta as the viewing axis and confirms the epitaxial relation between the Ta film and the sapphire substrate. The schematic in Figure 2c further illustrates the crystallographic orientation of the Ta film and the sapphire substrate. Laue pattern (Figure S3, Supporting Information) further confirms the epitaxial relation between Ta film and sapphire.

**Figure 3:**
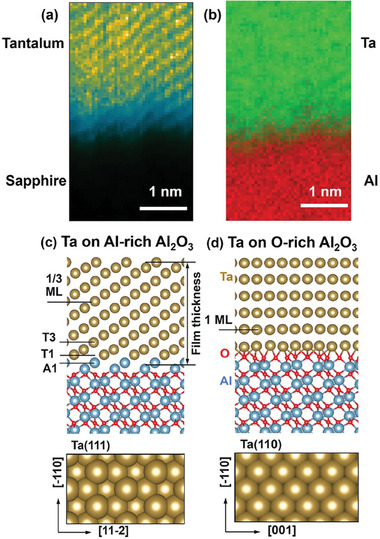
a,b) HAADF‐STEM false colored mass contrast imaging analysis of the Ta film on the metal/substrate interface and the intermixing of the Al‐Ta shows the degree of interfacial broadening. c,d) Structures of Ta/Al_2_O_3_ interfaces obtained using ab initio simulations: Ta(111) terminated film formed as a 12 continuation of the Al stacking sequence and Ta(110) terminated film stabilized by Ta‐O interactions on the Al‐rich and O‐rich substrates, respectively. The number density of Ta atoms per atomic plane in the O‐rich case is approximately three times that in the Al‐rich case.

To correlate and confirm the existence of the interface layer (i.e., M‐I‐S layer) between the M‐S layers, chemical mapping at the atomic scale was employed using HAADF‐STEM with EELS. **Figure** **3** shows the HAADF‐STEM false‐colored mass contrast imaging analysis for atomic scale measurements in the M‐S interface region. Although the Ta film exhibits high epitaxial quality with the sapphire substrate, the M‐S interface appears broader rather than sharp. Additionally, we observe the mixing of Ta (green) and Al (red) in the interface layer. EELS scans were performed in multiple areas across the samples to understand this region better, as shown in Figure S4a (Supporting Information). The energy of the Ta L_3_ edge (9.8 keV) is not suitable for measurements due to its low S/N ratio^[^ ^15^ ^]^ Therefore, in this case, we performed EELS measurements in the energy region of 1400 to 1800 eV, which provides information about the Ta M_4,5_ edge and Al K‐edge. EELS analysis with a pixel size of ≈0.05 nm on the broad interface layer confirmed a uniform intermixing of Ta, Al, and O atoms (Figure S4b,c, Supporting Information). The thickness of this M‐S interface layer was determined to be 0.65 ± 0.05 nm. This analysis is consistent with the results obtained from XRR measurements. These thickness values are close to the resolution limit for each method. The difference in the thickness values can be ascribed to the indistinct boundaries of the interface layer indicated by the HAADF‐STEM measurements versus the slab‐based modeling employed from the XRR fitting (0.205 ± 0.047 nm).

In addition, the chemical profile of the Ta film across the surface region is quantified based on the VEXPS technique (Figure S5, Supporting Information) to further correlate the surface oxide parameters (M‐A interface) with the fitted XRR values. By varying the incident photon energy from low (690 eV) to high (4500 eV) (see Figure S5a‐f, Supporting Information), the kinetic energy of emitted photoelectrons can be varied, thereby providing control over mean free paths and resulting in the measurement of photoelectrons at depths up to ≈12 nm. The detailed description used for fitting the Ta 4f spectra, such as background subtraction, error calculations, and mixed suboxide features, can be found in our previous work^[^ ^14^ ^]^ Figure S5g,h (Supporting Information) shows that beneath a thickness of 2 to 2.5 nm (from the top), most of the Ta film exists in the pure metallic state. This depth profiling result from VEXPS measurement is consistent with the previously published works on native Ta film.^[^ ^14^, ^17^ ^]^ The thickness obtained for Ta_2_O_5_ (Ta^5+^) using VEXPS measurements is ≈1.676 ± 0.034 nm. Moreover, this variation in thickness can be attributed to the growth of native oxide layer over time since the VEXPS data was collected sometime after the synchrotron XRR experiments. Given this and our previous VEXPS results of freshly deposited films, we can also confirm the self‐passivating nature of the oxide layer formed at the M‐A interface.

To further our insight into the connection between the structure, stability, and electronic properties of Ta/Al_2_O_3_ heterojunctions, DFT modeling was utilized. The two types of Ta/Al_2_O_3_ interfaces formed by depositing Ta metal on the Al‐rich and O‐rich Al_2_O_3_ (0001) surfaces, respectively, are shown in Figure 3c,d. In the case of the Al‐rich surface, atoms of the first Ta monolayer (T1) were found to be most stable at the hollow sites of the Al bilayer. Accordingly, the first Ta plane has the same number density and atomic arrangement as the topmost Al plane, resulting in a seamless Ta/Al interface. Additional Ta atoms also occupy the hollow sites, thus forming a BCC Ta film terminated with the Ta (111) surface. This structure (Figure 3c) exhibits slight variations in the calculated interplane distances for the topmost Al–Al (0.783 Å), interfacial Al–Ta (1.163 Å), and Ta–Ta (0.898 Å on average) planes. Based on the experimental lattice parameters of the bulk Al_2_O_3_ and Ta, the film is subjected to an ≈1.7% uniform tensile strain. A similar strain value (≈2.1%) is obtained if the computed lattice parameters are used instead.

In the case of O‐rich Al_2_O_3_ (0001), the terminating surfaces contain the full oxygen bilayer, which corresponds to the average formal charge state of the surface oxygen species of O^−^. Upon relaxation, this surface undergoes disproportionation of the electron charge between the surface oxygen species resulting in the formation of peroxy (O_2_^2−^) and superoxide (O_2_^−^) ions in addition to O^2−^. A single monolayer of Ta metal deposited on such a surface provides enough electrons to fully complete the O 2p shells of all surface oxygen species. The resulting Ta ions are distributed over the surface to form a (110) surface of the BCC Ta lattice, as shown in Figure 3d. The Ta film is subjected to 1.7% tensile strain in the [‐110] direction. However, there is no obvious lattice match along the [001] direction. Inspection of the STEM images of the Ta/Al_2_O_3_ interfaces^[^ ^9^ ^]^ suggests that the periodic structure of the interface is determined by the 7:8 ratio of the Ta to Al_2_O_3_ lattice spacings in the ←112> and <11‐20> directions, respectively. Due to the constraints of the periodic model approach, we considered the supercells with the ratios of 5:6 and 8:9 (not shown), which correspond to ≈4.2% tensile and 1.4% compressive strains of Ta film relative to the experimentally observed systems and result in similar structures.

**Figure 4:**
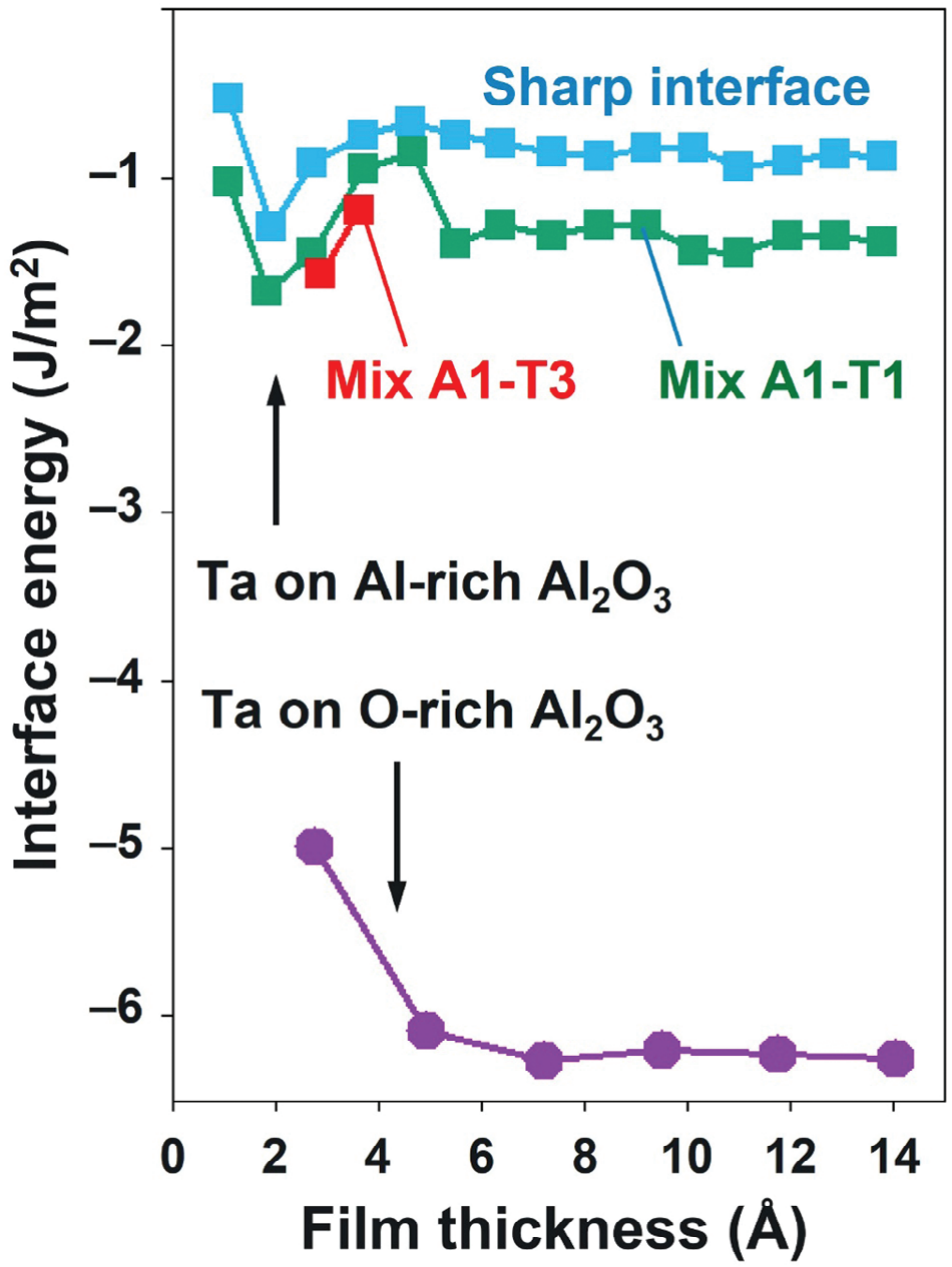
Interface energy as a function of Ta film thickness for the sharp and intermixed configurations formed by swapping Al and Ta atoms in planes A1, T1, and T3 indicated in (Figure 3c).

To evaluate the thermodynamic stabilities of these Ta films on the Al_2_O_3_ substrate (**Figure** **4**; Figure S6, Supporting Information), we first compared the surface energies of the relaxed (110) and (111) surfaces (2.33 and 2.74 J m^−2^, respectively) and the energies required to strain Ta slabs terminated with these surfaces to match the supercell parameters of the Al_2_O_3_ substrate: ≈25 meV per atom in both cases. Then, the interfacial energies of the films were calculated relative to the energies of pure O‐rich and Al‐rich alumina surfaces and the cohesive energy of the bulk Ta and corrected for the contributions due to the substrate‐induced strain and energy of the exposed surface, resulting in −6.25 and −0.88 J m^−2^, respectively. These values correspond to the limiting cases of the fully oxidized and fully reduced surfaces. In general, interfacial energies and structures of these interfaces are defined by the oxygen content and spatial distribution of these oxygen species over the surface.

**Figure 5:**
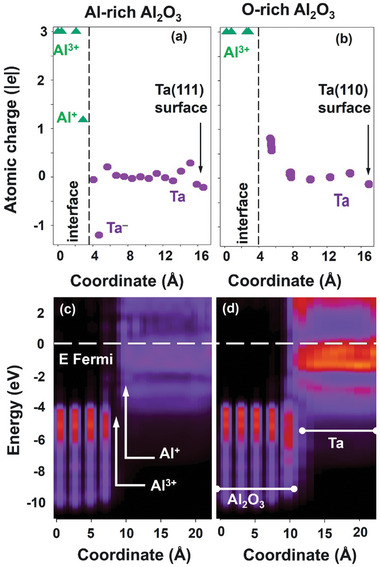
a,b) Charge distributions in selected Ta/Al_2_O_3_ systems represented by layer‐averaged Bader atomic charges for Ta on the Al‐rich (a) and O‐rich (b) Al_2_O_3_. Vertical dashed line represents the Ta/Al_2_O_3_ interface. c,d) Heatmap representations of one‐electron DOS calculated for the Ta on the Al‐rich (c) and O‐rich (d) Al_2_O_3_ systems and projected on the Al, O, and Ta atomic planes. The energy scales are aligned so that Fermi energy is at 0 eV. The difference in the Ta DOS intensity in (c) and (d) is due to different Ta stacking sequences along the out‐of‐plane direction.

The charge distribution for both types of the Ta/Al_2_O_3_ structures is shown in **Figures** **5** and S7 (Supporting Information). In the case of the Al‐rich Al_2_O_3_ substrate, the electron charge is transferred from the outermost Al plane to the second from the interface Ta plane, resulting in the interfacial dipole in the out‐of‐direction (Figure 5a). For comparison, in the case of the O‐rich Al_2_O_3_ substrate, Ta atoms of the first monolayer donate their electrons to the outermost oxygen plane, resulting in the interfacial dipole opposite to that found for the Al‐rich case (Figure 5b). This charge redistribution is short ranged as the Ta atoms beyond the first interfacial plane are nearly neutral.

To assess the width of the region affected by such electron redistribution, we calculated the interfacial energy as a function of the film thickness (Figure 4). In the O‐rich case, this energy is the largest for the first Ta monolayer and nearly constant after that, consistently with the charge distribution. In contrast, in the Al‐rich case, the interfacial energy shows noticeably larger variations with the film thickness indicating its sensitivity to the details of the atomic positions across the film. Given that Al metal cohesive energy (3.39 eV atom^−1^) is much smaller than that of the bulk Ta (8.10 eV atom^−1^)^[^ ^30^ ^]^ it can be expected that the outer plane of Al (A1 in Figure 3c) dissolves into the Ta films at the initial stages of the film deposition. In particular, we found that deposition of the first 1/3 ML of Ta atoms on the Al‐rich surface results in a spontaneous in‐diffusion of these atoms so that they occupy the space between two Al planes (see Figure S8, Supporting Information).

To assess the effect of such Ta‐Al intermixing, we considered several configurations in which Al atoms of the outermost substrate plane (denoted as A1) were swapped with Ta atoms in the 1st, 2nd, or 3rd place from the interface (denoted as T1, T2, and T3, respectively). The A1‐T1 swap was found to stabilize the Ta film by as much as 0.5 J m^−2^ for all film thicknesses and the A1‐T3 swap has an even larger stabilizing effect for up to 4/3 ML Ta coverage (Figure 4), while the A1‐T2 swap had a destabilizing effect only (not shown). These calculations suggest that excess Al indeed dissolves into the Ta film forming a buffer layer, consistent with the experimentally observed transition layer. Furthermore, charge distribution for the intermixed configurations (see Figure S5, Supporting Information) in the case of the thick film shows the sequence of positively and negatively charged layers formed by the Al and Ta species respectively which stretches at least 0.6 nm into the Ta film, which qualitatively agrees with the width of the buffer layer estimated from the experimental observations.

Finally, to analyze the electronic structure of the Ta/Al_2_O_3_ interfaces, we plotted the one‐electron density of states projected on the atomic planes for selected cases (Figure 5c,d; Figure S9, Supporting Information). In the case of the ideal Ta on the Al‐rich Al_2_O_3_, there is a noticeable presence of metal states in the Al_2_O_3_ band gap region near this interface (Figure 5c). As this interface is stabilized via Al‐Ta intermixing, the DOS near Al in the Ta film is severely depleted even though the Ta‐Al film is nominally metallic. We attribute this effect to the charge disproportionation between the Al and Ta species resulting in positively charged Al species as indicated in Figure 5. Whereas, in the case of the O‐rich substrate, the interface is electronically abrupt and expected from the well‐ordered structure of the interface (Figure 5d). Overall, the DFT modeling, corroborated by HAADF‐STEM analysis, confirm that our Ta films are grown on Al‐rich Al_2_O_3_ surface.

**Figure 6:**
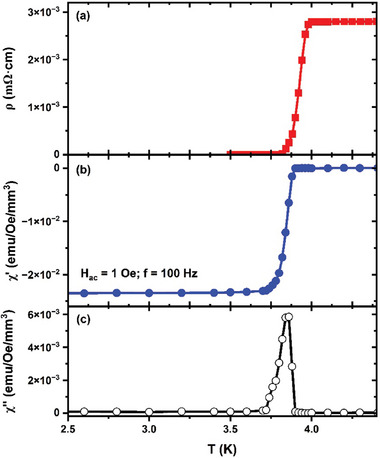
a) Temperature dependent resistivity of the 30 nm Ta film. b,c) The real and imaginary component of ac magnetic susceptibility of the same film under a drive amplitude of 1 Oe and a frequency of 100 Hz.

With well‐characterized Ta interfaces and theoretically predicted interface structure, which is usually metallic but has charge depleted Al, it remains to be understood how this M‐S interface layer can affect the physics governing qubit performance. To address this, low‐temperature transport measurements were conducted to determine the effects on the superconducting properties of the Ta film due to the existence of an interface layer between the metal and substrate layers. **Figure** **6**a shows the resistivity measured as a function of temperature for the Ta film, which has a superconducting transition temperature *T*_c_ of 3.84 K and mid‐point transition *T*_c_ of 3.93 K, with a residual resistivity ratio (RRR) value (*ρ* (4 K)/ *ρ* (300 K)) of 4.98. This *T*_c_ value is slightly lower than those reported in previous works for a pure BCC structure Ta film (*T*_c_ = 4.0 K to 4.4 K).^[^ ^17^, ^29^, ^31^ ^]^ However, several previous studies have also reported T_c_ values of BCC structure Ta films to be less than 4 K.^[^ ^32^, ^33^, ^34^ ^]^ Table S1 (Supporting Information) summarizes some of the previous studies performed on Ta‐based superconducting films on various sapphire and silicon substrates. Figure 6b,c shows the AC magnetic susceptibility measurements of the Ta film under a drive amplitude of 1 Oe. The magnetic field was applied parallel to the film's surface. The mid‐point *T*_c_ of the *χ*“ and the peak location of *χ*” is at 3.86 K, similar to the *T*_c_ obtained from the resistivity measurement. The lower T_c_ could be attributed to the presence of the M‐S interface layer that we experimentally observed and confirmed its possible existence through DFT modeling. Alternatively, it could be due to the defects present in the films. This, in turn, resulted in a lower RRR value, indicating the film exhibits type‐II superconductivity.

This multimodal study provides concrete evidence of an interface layer between the M‐S layers, identified using XRR and HAADF‐STEM techniques. Additionally, DFT modeling reveals that the atomic and electronic properties of Ta/Al_2_O_3_ heterojunctions are significantly influenced by the surface composition of Al_2_O_3_. Specifically, O‐rich surfaces promote the formation of peroxide or superoxide ions, while Al‐rich surfaces result in considerable Ta‐Al intermixing at the interface. These variations critically affect the thermodynamic stability and electronic characteristics of the heterojunctions, potentially affecting the T_1_ time of qubits.

Generally, material loss research is currently focused on three factors in order to improve qubit lifetimes: dielectric properties of the substrates, Josephson junction losses, and interface losses^[^ ^35^ ^]^ Previous 3D cavity measurements have shown that the loss tangent of a 5 nm thick oxide is ≈0.1, which exceeds the losses at the metal/substrate interface. However, Wisbey et al. found that TLS loss is influenced by both the M‐S interface and surface roughness^[^ ^36^ ^]^ Recently, McFadden et al. identified the Ta/sapphire interface as the source of loss in epitaxial Ta(111) films on Al₂O₃(0001), with quality factors improving over an order of magnitude by adding a 5 nm Nb interlayer or plasma‐treating the substrate^[^ ^37^ ^]^ Looking forward, it is not unreasonable to suggest that studies involving bulk loss in resonators material will become more important when M‐S and M‐A interfaces cannot be further engineered to have significant impact on TLS loss. In that light, the presented DFT findings elucidate the growth mechanism for Ta (110) or Ta (111) orientations on different Al_2_O_3_ (0001) terminated surfaces. This insight into controlling Ta film orientations could help in optimizing surface and interface properties, ultimately contributing to improved qubit lifetimes and reduced TLS losses. Moreover, the effects of mixed surface terminations of sapphire on the crystallography of the deposited Ta film remain are not yet fully understood^[^ ^38^ ^]^ Specifically, the presence of mixed crystalline habitats and the consistency of the imposed strain across bulk Ta films in the 150 to 300 nm thickness range need further clarification. Additionally, more investigation is required to understand the relaxation process of this strain to its nominal value and its impact on the film's morphology and roughness^[^ ^9^ ^]^ The current study indicates that although the M‐S interface may not directly contribute to TLS, the M‐S interface plays a crucial role in determining the characteristics of the resulting film. We have demonstrated several experimental methods to investigate this region, and our DFT results suggest that previously reported record‐breaking lifetimes are associated with O‐terminated sapphire and Ta(110) oriented films.

## 3 Conclusion 

This study demonstrates that the epitaxial growth of a Ta (111) thin film on a c‐plane (0001) sapphire leads to the formation of an interface layer at the metal‐substrate interface and that the structure of this layer is potentially controlled at the atomic level by tuning the sapphire surface termination. Using a combination of synchrotron X‐ray reflectivity and scanning transmission electron microscopy, we investigated the quality of the metal‐substrate interface layer. XRR revealed the unexplored interface layer at the M‐S interface is ≈0.205 ± 0.047 nm. HAADF‐STEM measurements confirmed this interfacial layer, indicating a thickness of 0.65 ± 0.05 nm. The difference in thickness can be attributed to the indistinct boundaries of the interface layer observed in HAADF‐STEM. The structure, stability, and electronic properties of Ta/Al_2_O_3_ heterojunctions examined by DFT modeling further confirmed the possible intermixing at the M‐S interface. This multimodal approach, employing various material characterization techniques, enhances our understanding of the interface layer between the metal and substrate. These insights can help to pave the way for controlling this interface layer and hence improving the coherence time of superconducting qubits.

## Conflict of Interest

The authors declare no conflict of interest.

## Disclaimer

Certain commercial equipment, instruments, or materials are identified in this paper in order to specify the experimental procedure adequately, and do not represent an endorsement by the National Institute of Standards and Technology.

## Author Contributions

A.k.A., A.L.W., and A.B., conceived the idea and designed the experiments. A.k.A., A.B., J.J.S., and V.S. performed synchrotron‐based XRR experiments. M.L., and C.Z. were responsible for material synthesis and J.Y., and Q. Li for low‐temperature transport measurements. R.C., J.M., and K.K. conducted electron microscopy experiments. A.k.A. and D.N. performed lab‐based XRR measurements. P.V.S. carried out theoretical calculations. A.k.A., C.Z., and C.W. performed synchrotron‐based XPS experiments. S.L.H., N.P. L., Y.Z., M.L., P.V.S., A.L.W., and A.B. supervised the project. A.k.A., P.V.S., A.L.W., and A.B. drafted the manuscript. All authors participated in editing the manuscript.

## Supporting information



Supporting Information

## Data Availability

The data that support the findings of this study are available from the corresponding author upon reasonable request.
