# Supplementary material for: Revealing the Origin and Nature of the Buried Metal‐Substrate Interface Layer in Ta/Sapphire Superconducting Films
Source: Adv Sci (Weinh). 2025 Feb 19;12(17):2413058. doi: 10.1002/advs.202413058 (PMC12061325; doi:10.1002/advs.202413058)
Supplement: Supplementary file 1 — Supporting Information [file ADVS-12-2413058-s001.docx]

**Supporting information for**

**Revealing the Origin and Nature of the Buried Metal-Substrate Interface Layer in Ta/Sapphire Superconducting Films**

Aswin kumar Anbalagan ^1, *^, Rebecca Cummings ^2^, Chenyu Zhou ^3^, Junsik Mun ^2,3^, Vesna Stanic ^4^, Jean Jordan-Sweet ^4^, Juntao Yao^2,5^, Kim Kisslinger ^3^, Conan Weiland ^6^, Dmytro Nykypanchuk ^3^, Steven L. Hulbert ^1^, Qiang Li ^2,7^, Yimei Zhu ^2^, Mingzhao Liu ^3^, Peter V. Sushko ^8, *^, Andrew L. Walter ^1, *^, and Andi M. Barbour ^1, *^

^1^ National Synchrotron Light Source II, Brookhaven National Laboratory, Upton, New York 11973, USA.

^2^The Condensed Matter Physics and Materials Science Department, Brookhaven National Laboratory, Upton, New York 11973, USA.

^3^ Center for Functional Nanomaterials, Brookhaven National Laboratory, Upton, New York 11973, USA.

^4^ IBM T. J. Watson Research Center, 1101 Kitchawan Road, Yorktown Heights, New York 10598, USA.

^5^Department of Materials Science and Chemical Engineering, Stony Brook University, Stony Brook, New York 11794, USA.

^6^Material Measurement Laboratory, National Institute of Standard and Technology, Gaithersburg, Maryland 20899, USA.

^7^Department of Physics and Astronomy, Stony Brook University, Stony Brook, New York 11794, USA.

^8^Physical and Computational Sciences Directorate, Pacific Northwest National Laboratory, Richland, Washington 99354, USA.

*Corresponding authors: Aswin kumar Anbalagan ([aanbalaga1@bnl.gov](mailto:aanbalaga1@bnl.gov)); Peter V. Sushko ([peter.sushko@pnnl.gov](mailto:peter.sushko@pnnl.gov)); Andrew L. Walter ([awalter@bnl.gov](mailto:awalter@bnl.gov)); and Andi M. Barbour ([abarbour@bnl.gov](mailto:abarbour@bnl.gov)).

1. **Experimental**
   1. **Thin film preparation**

Tantalum thin film sample was prepared by RF magnetron sputtering on a 2-inch C-cut sapphire (0001) (CrysTec, single-side polished) at a substrate temperature of 750 °C in an AJA Orion Sputtering System equipped with A315-UHV sputtering sources. The base pressure of the sputtering chamber was maintained at 10^-6^ Pa. Before loading the sapphire wafer into the deposition chamber, it was cleaned with a piranha solution made by mixing a commercial 30% hydrogen peroxide solution and 98% sulfuric acid in a volumetric ratio of 1:2, followed by rinsing with DI water. The Ta target (99.98 % purity) was procured from AJA International, Inc. The sputtering was performed at a working pressure of 1.6 Pa, with Research Grade Ar (Airgas) as plasma gas and a sputtering power of 100 W (RF).

**1.2 Materials characterization**

Synchrotron-based X-ray reflectivity (XRR) measurements were performed using 8.6 keV energy on the 06-BMM beamline operated by the National Institute of Standards and Technology (NIST) at the National Synchrotron Light Source (NSLS-II), Brookhaven National Laboratory (BNL). For these measurements, a Si (111) monochromator was used, which has an energy resolution of 1.3 × 10^-4^ ΔE/E. The exit slit size at the Mythen detector was 0.12 mm (V) × 1 mm (H) before the reflected signal was collected. Background subtraction for the reflectivity measurements were performed by collecting pixel zero (60 pixels), integrating it from both sides, applying Gaussian smoothing (10 points), and then averaging 2 curves and subtracting them. Lab-based X-ray diffraction (XRD) and XRR measurements were collected on a Rigaku SmartLab II X-ray diffractometer using Cu Kα radiation. The fitting of these reflectivity measurements was performed using the genX software^1^. Transmission electron microscopy (TEM), high-angle annular dark-field (HAADF) with scanning tunning electron microscopy (HAADF-STEM), and electron energy loss spectroscopy (EELS) measurements were carried out on a JEOLARM-200F, with the sample prepared by focused ion beam (FIB) lift-off technique utilizing a dual beam SEM/FIB microscope (FEI Helios). Variable energy X-ray photoemission spectroscopy (VEXPS) measurements were performed at the SST-2 beamline operated by the NIST at NSLS-II, BNL. The energy of the incident X-ray beam was varied from 690 eV (soft X-ray regime) to 4500 eV (tender X-ray regime). During the measurements, the beam's incident angle to the sample was kept at 10° and the takeoff angle of the photoelectrons to the detector was 80°. A gold standard was used for energy calibration. Transport measurements were performed by the four-probe inline method in a 14 Tesla Quantum Design physical property measurement system (PPMS). The magnetic ac susceptibility measurements were measured in a Quantum Design magnetic properties measurement system (MPMS) with a SQUID (superconducting quantum interference device) magnetometer.

**1.3 Density functional theory (DFT) modeling**

The structure, stability, and electronic properties of Ta/Al_2_O_3_ heterojunctions were analyzed using the periodic slab model and two types of terminating Al_2_O_3_ (0001) surfaces: Al-rich and O-rich ones. Ta films, commensurate with either Al-rich or O-rich terminations of Al_2_O_3_ (0001), were deposited on both sides of the Al_2_O_3_ slab to form equivalent terminating Ta surfaces. The calculations were performed using the VASP package ^2,3^ and Perdew-Burke-Ernzerhof (PBE) ^4^ exchange-correlation functional. The projector-augmented-wave potentials were used to approximate the effect of the core electrons ^5^.

The plane-wave basis set cutoff was set to 500 eV. The convergence of total energies with respect to the plane-wave basis set cutoff energy was examined for the Ta film deposited on the Al-rich Al_2_O_3_ as shown in **Figure S6.** The total energy convergence criterion was set to 10^–5^ eV. Bader charge population analysis was used to analyze the charge density redistribution ^6,7^. The lateral supercell parameters were based on the bulk Al_2_O_3_ hexagonal lattice parameter (4.80813 Å) pre-calculated using the crystallographic cell and 5×5×2 k-mesh. The total energies were minimized with respect to all internal coordinates. The adsorption energies of Ta atoms were calculated relative to the Ta bulk cohesive energy. One-electron densities of states (DOS) were smeared out by convoluting band energies with Gaussian functions with the full width at a half-maximum of 0.1 eV.

The variations in the reflectivity fringe width between the synchrotron and laboratory-based XRR measurements at lower Qz can be attributed to the slow growth of the native oxide layer over time.


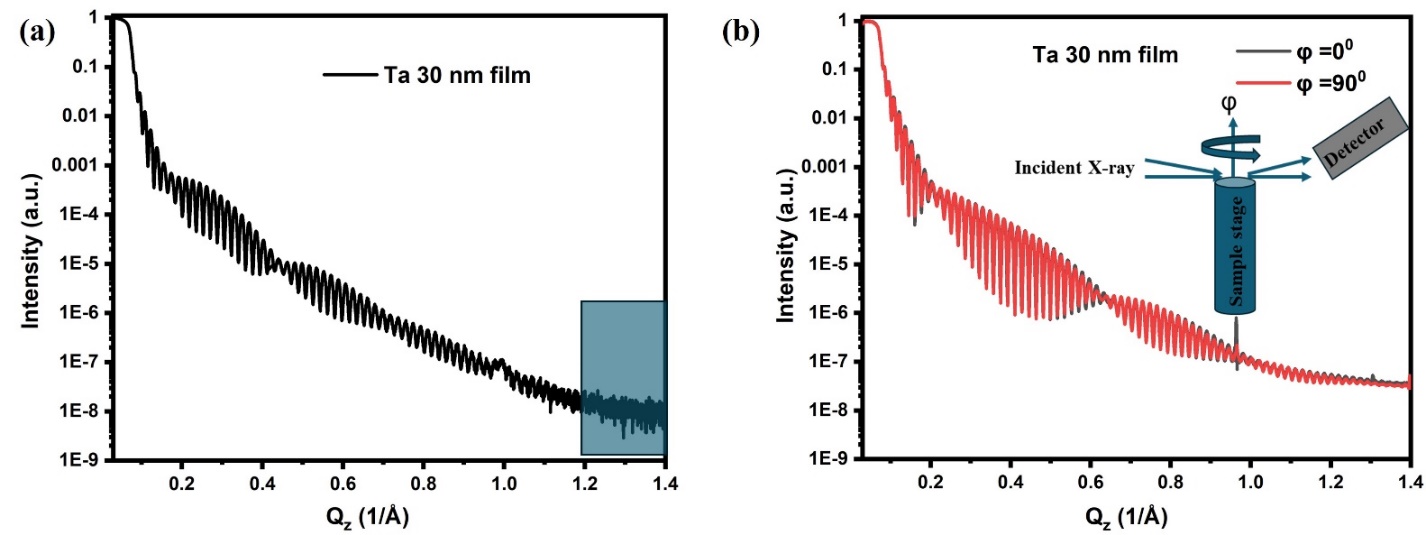


**Figure S1.** X-ray reflectivity of Ta film using (a) lab-based and (b) synchrotron-based tools. The shaded area in the lab-based XRR plot highlights the crucial range of Q_z_ values responsible for determining the information about the metal-substrate interface of the Ta film. The lab-based XRR measurements were performed later in time, closer to that of the data summarized in **Figure S5**. Fitting of the lab-based XRR data is consistent with a slightly thicker native oxide layer at the metal-air interface.


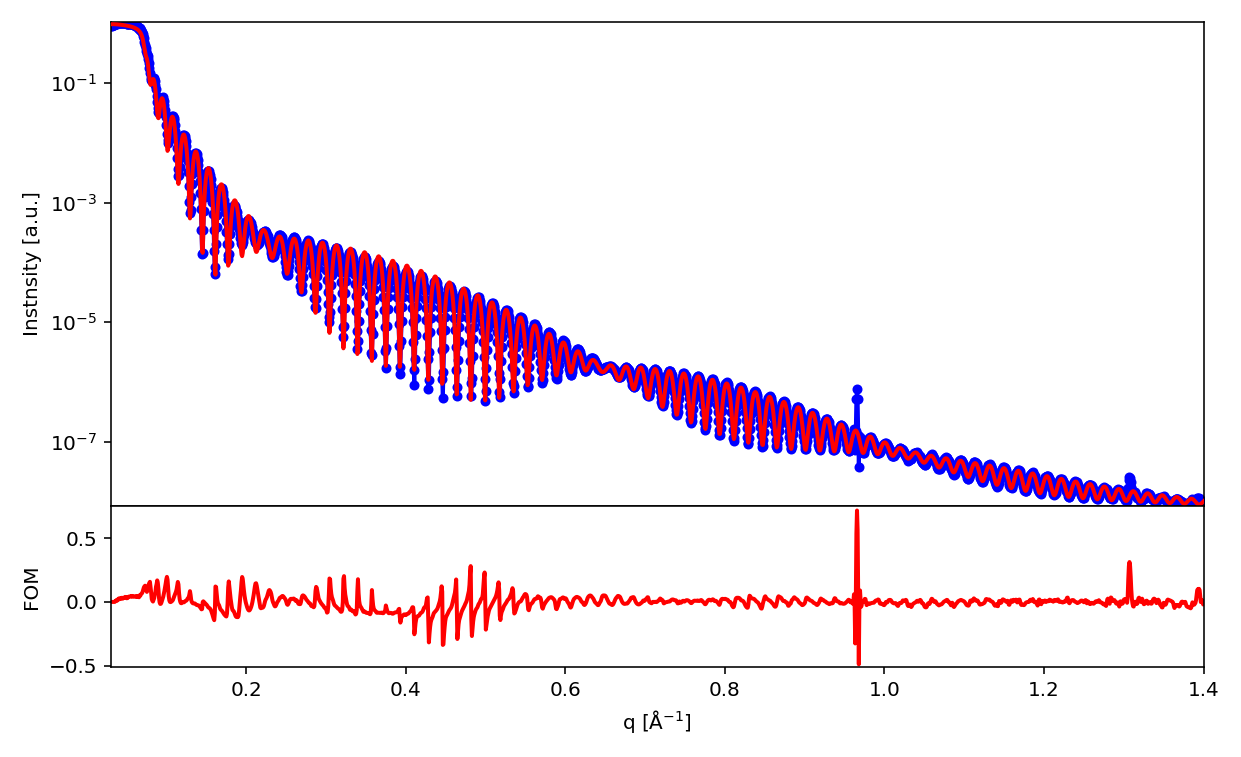


**Figure S2.** GenX fitting result of the synchrotron X-ray reflectivity measurements for the BCC Ta film on sapphire substrate. Model 4 with interface layers between both the Ta_2_O_5_/metal and substrate/metal layers.


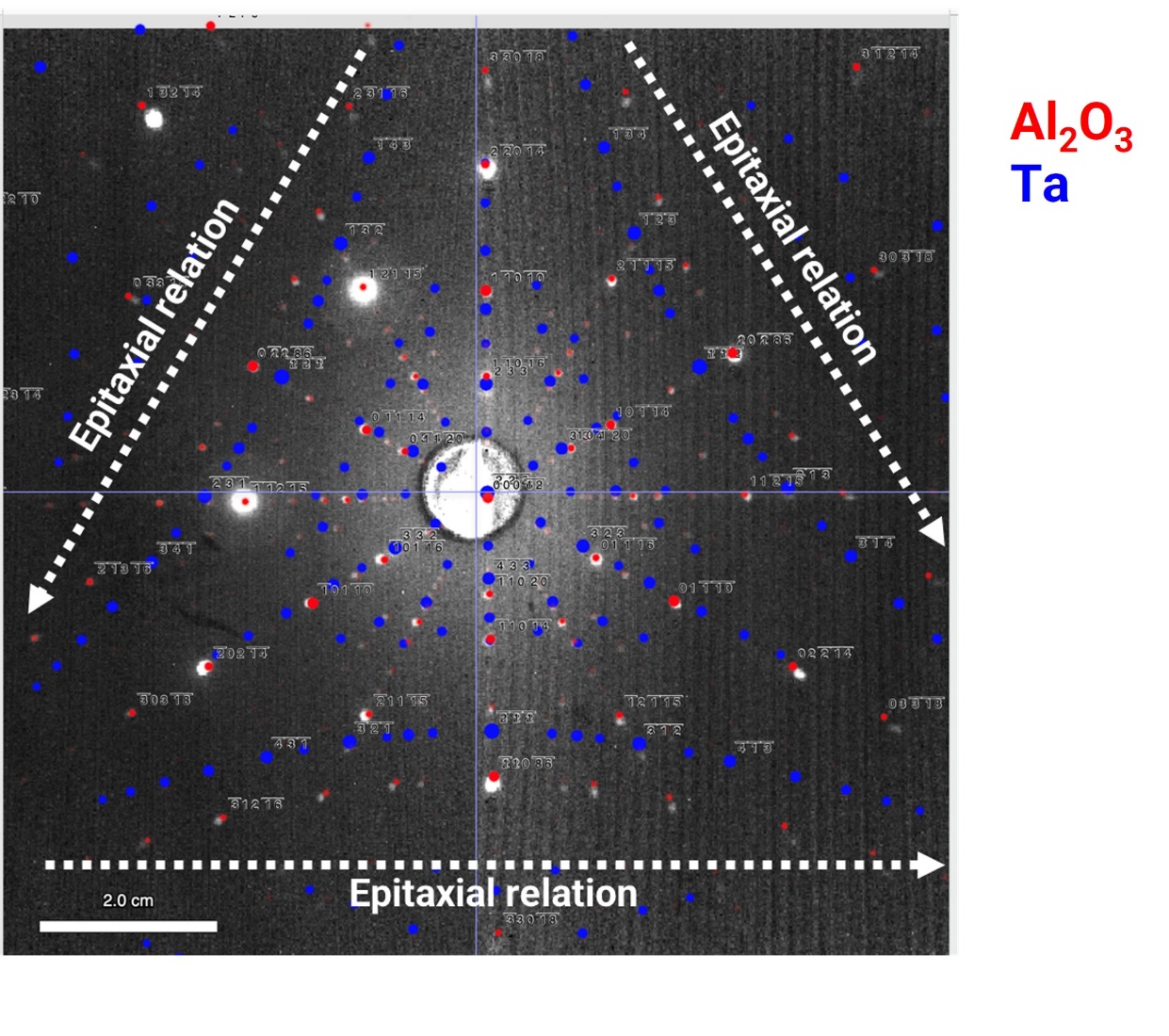


**Figure S3.** The Laue pattern illustrates the epitaxial relationship between the Ta film and the substrate. For phi=0 in the vertical scattering geometry, the incident X-rays come from the bottom (or top) of the page.

EELS scans were performed in multiple areas across the samples, as shown in **Figure S4a**, to better understand this region. **Figure S4b, c** shows the EELS scan of spots 1 and spot 2 measured in the energy region of 1400-1800 eV. These EELS measurements reveal the co-existence of both Ta and Al atoms at all measured spots, indicating the broad interface layer is uniform across the metal-substrate layer.


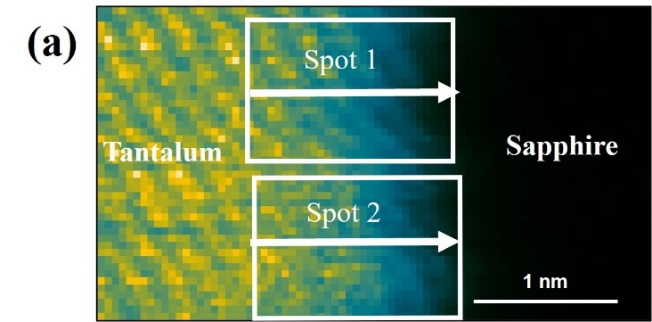


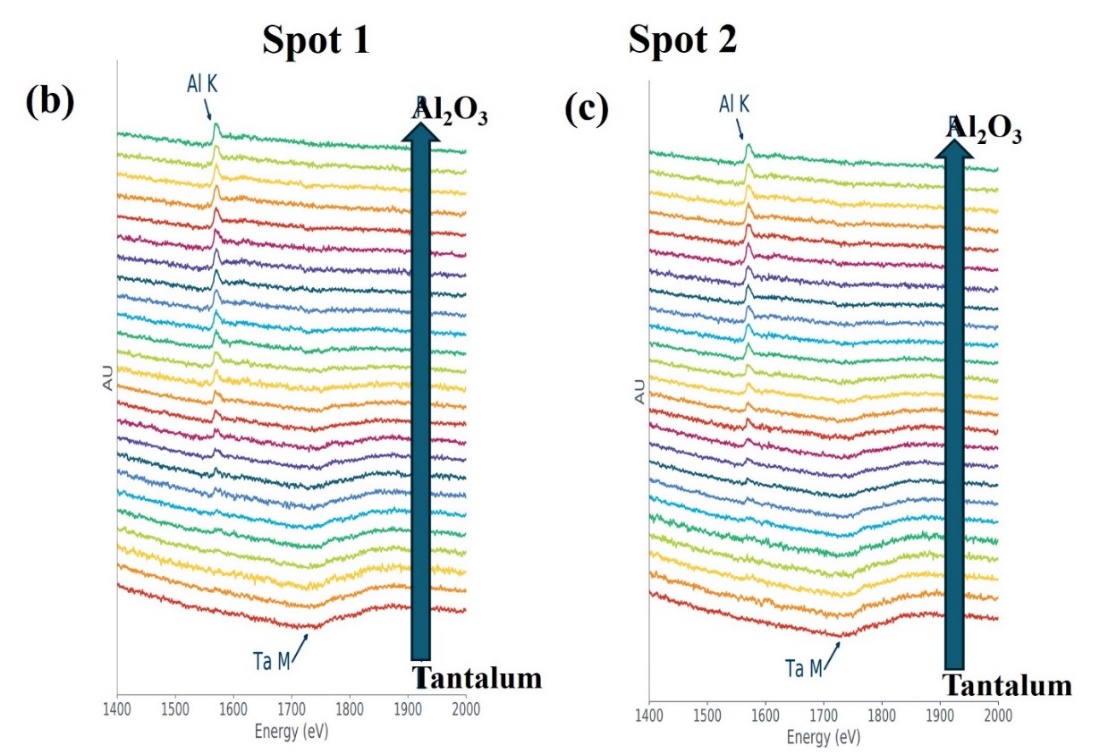


**Figure S4.** (a) HAADF-STEM false colored mass contrast imaging analysis of the Ta film on the substrate-metal interface and (b, c) the corresponding EELS scans of the highlighted regions Spot 1 and Spot 2. And the arrows denote the EELS scanning direction.

The VEXPS technique quantifies the chemical profile of the Ta film’s surface region, correlating the surface oxide parameters with fitted XRR values. By varying the incident photon energy from low (690 eV) to high (4500 eV) (**Figure S5 a-f**), the kinetic energy of emitted photoelectrons can be varied, allowing depth measurements up to about 10 nm. **Figure S5 g-h** shows that below a thickness of 2-2.5 nm from the top, most of the Ta film remains in a pure metallic state.


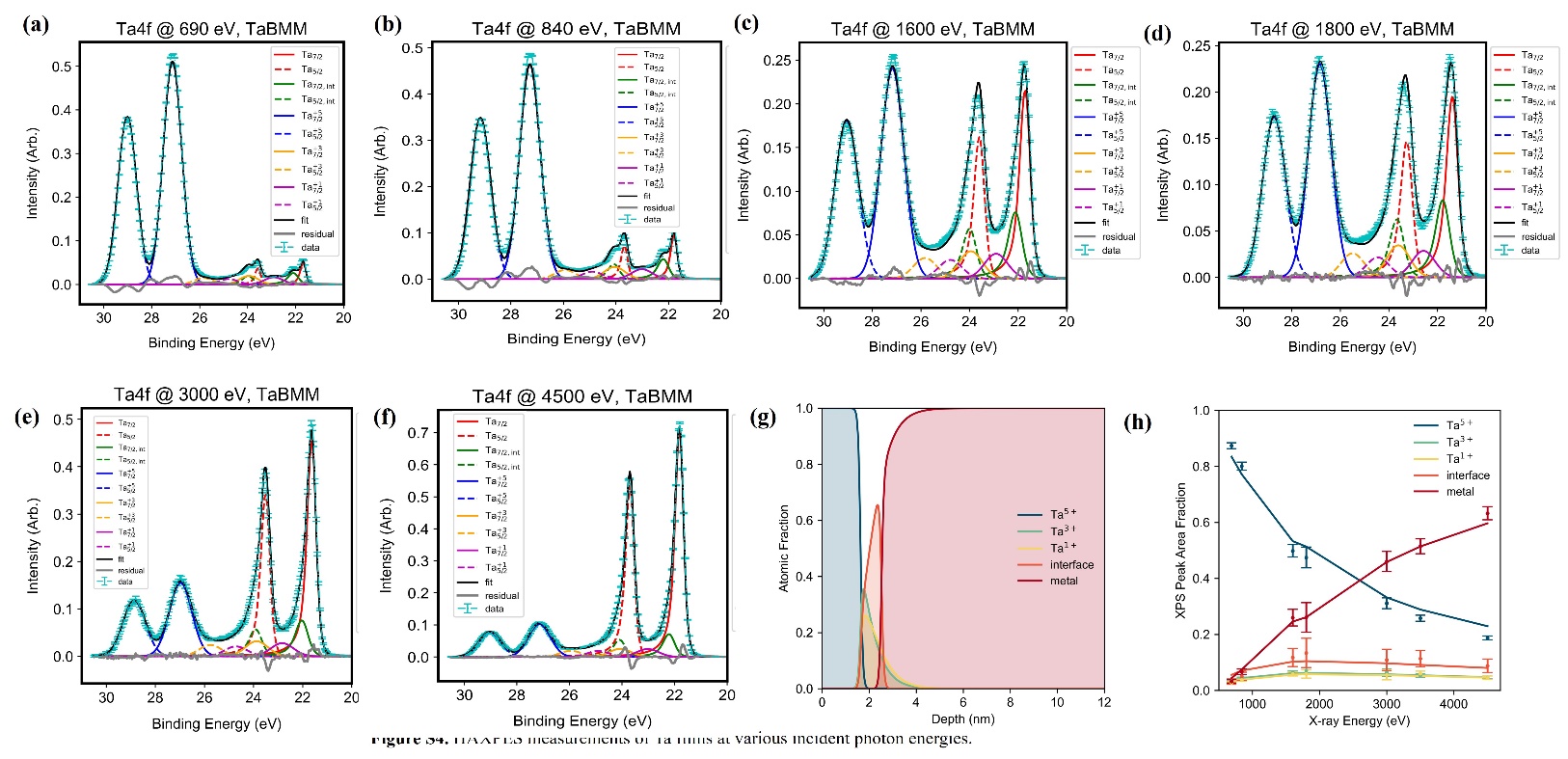


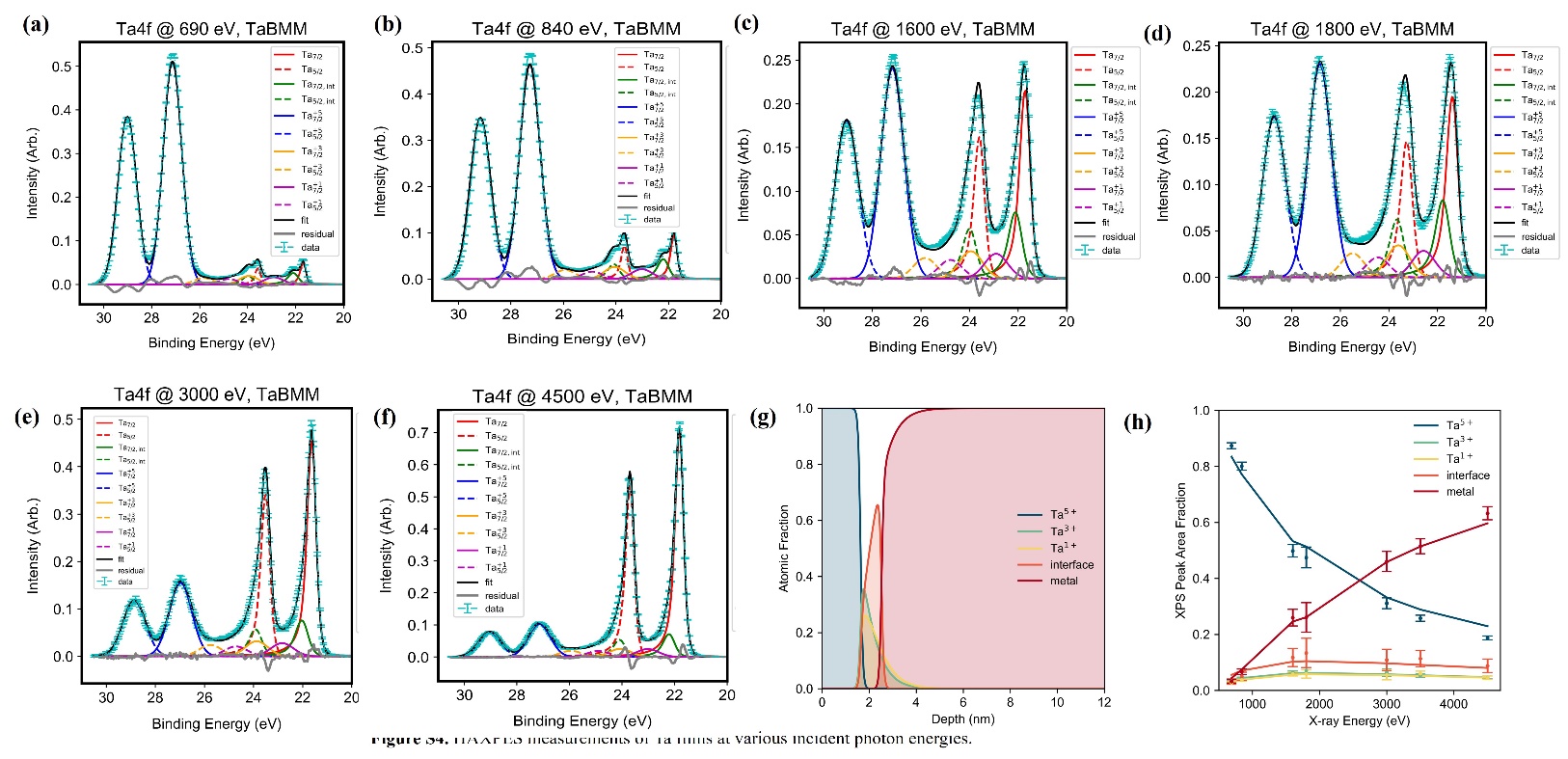


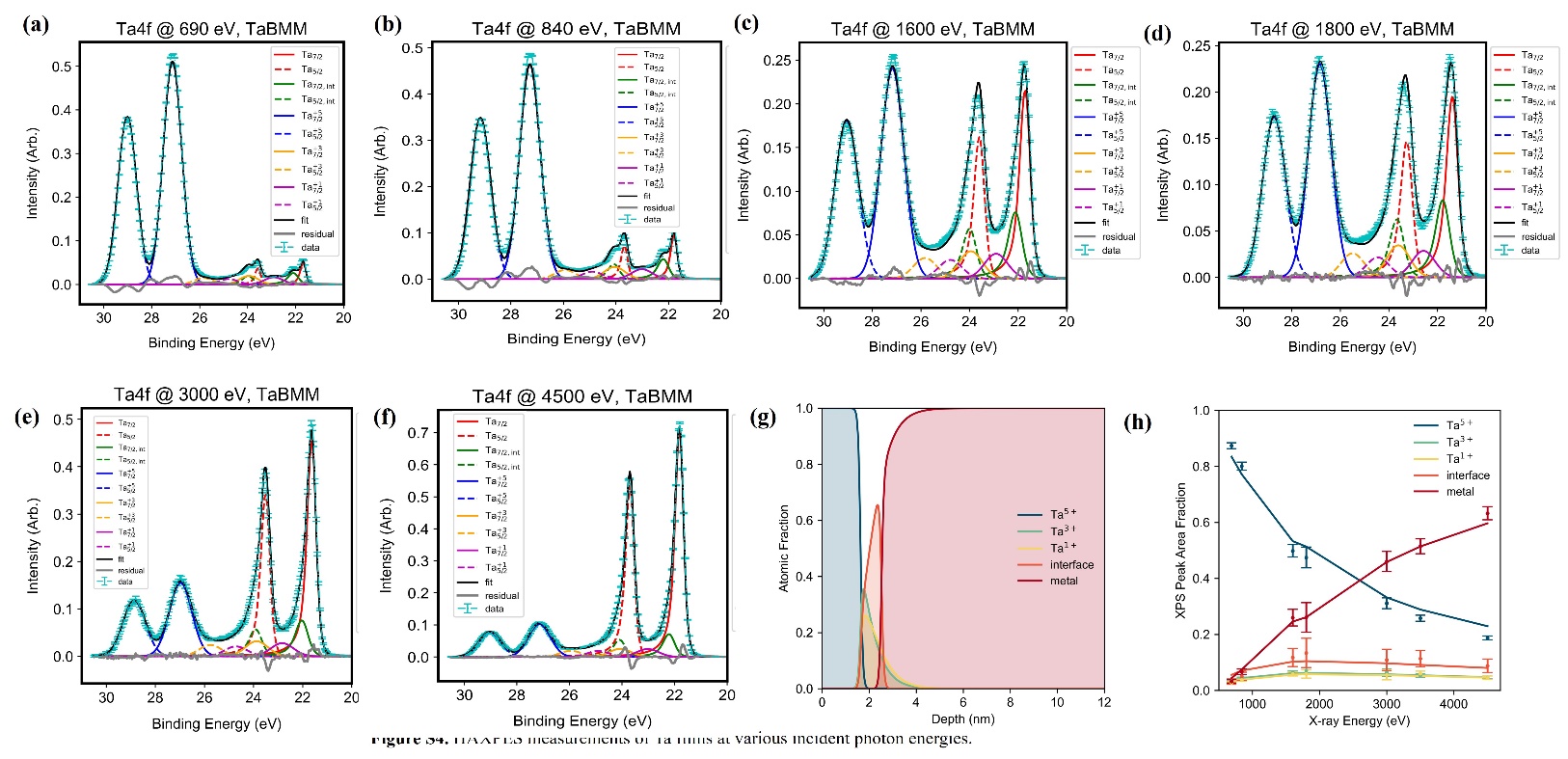


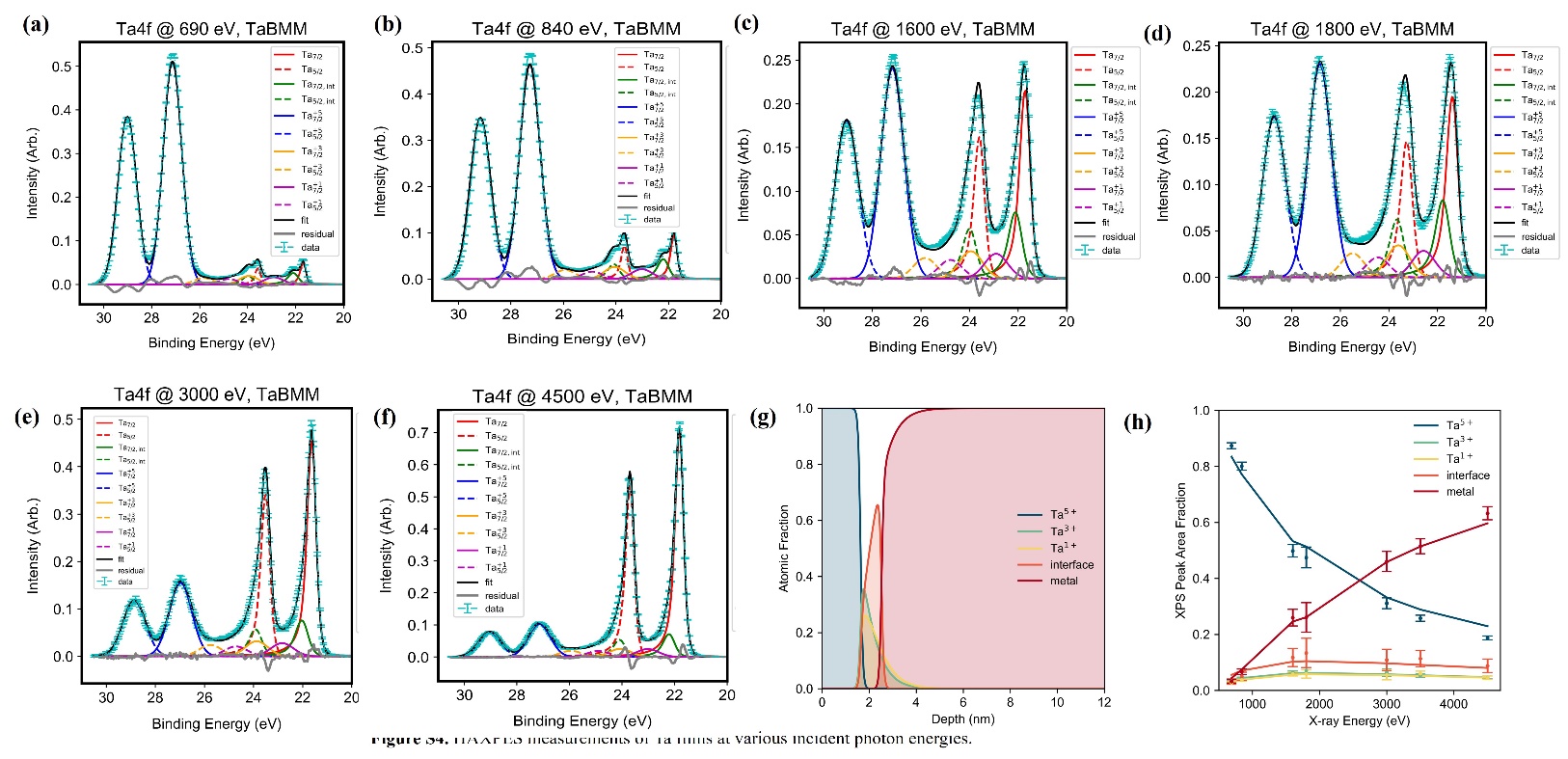


**Figure S5.** VEXPS measurements of Ta films at various incident photon energies.


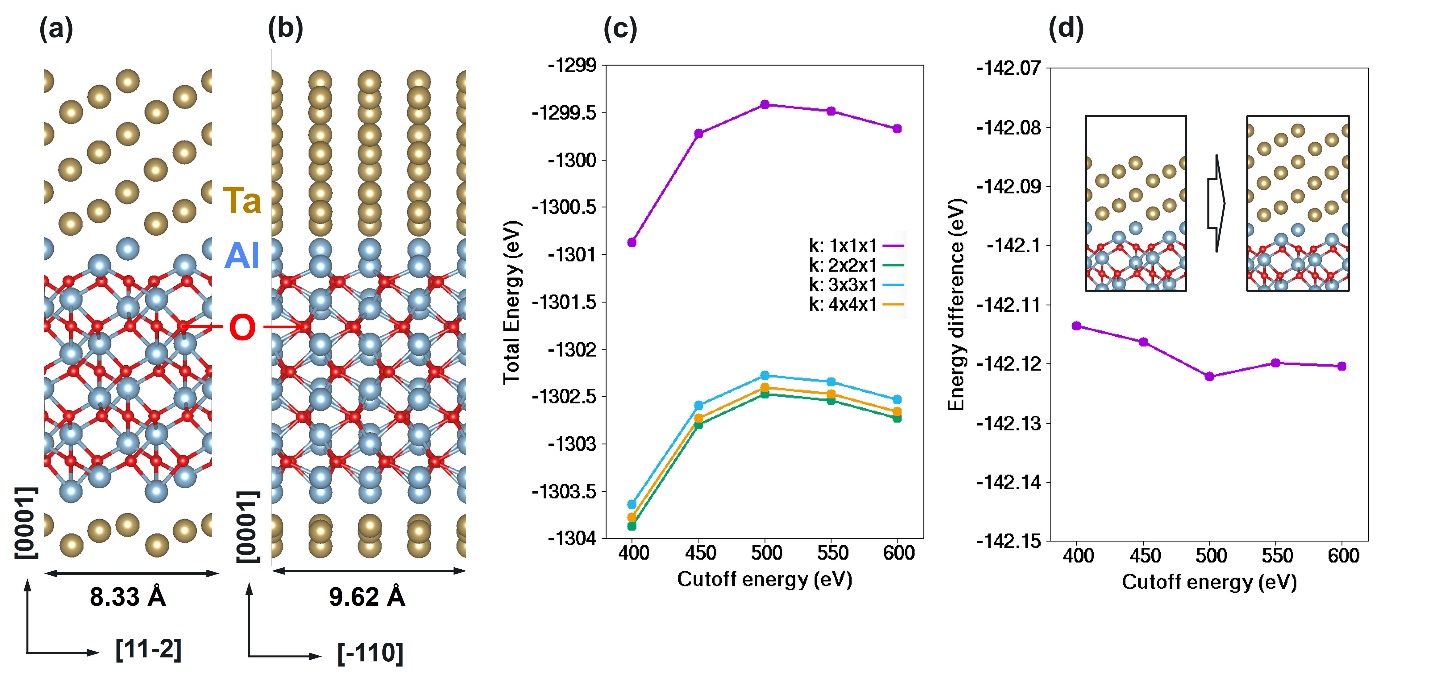


**Figure S6.** The convergence of total energies with respect to the plane-wave basis set cutoff energy was examined for the Ta film deposited on the Al-rich Al_2_O_3_, shown in panels (a) and (b). The bottom and top surfaces of the Al-terminated slab are capped with three and nine Ta(111) planes, respectively. (c) The total energies calculated for several Gamma-centered k-meshes. For k-meshes 2×2×1, 3×3×1, and 4×4×1, the total energies are within 0.25 eV (< 1.6 meV per atom). (d) Energy difference (ΔE) associated with increasing thickness of the Ta film from 6 to 9 Ta(111) panes. For the cutoff energies of 500-600 eV, ΔE values are within 2 meV interval, or within 0.7 meV per Ta(111) plane. Given the supercell lateral area (~80 Å^2^), the uncertainly of the binding Ta energies is ~0.15 mJ/m^2^, which is negligible in comparison to the interfacial energies (see **Figure 4** in the manuscript).


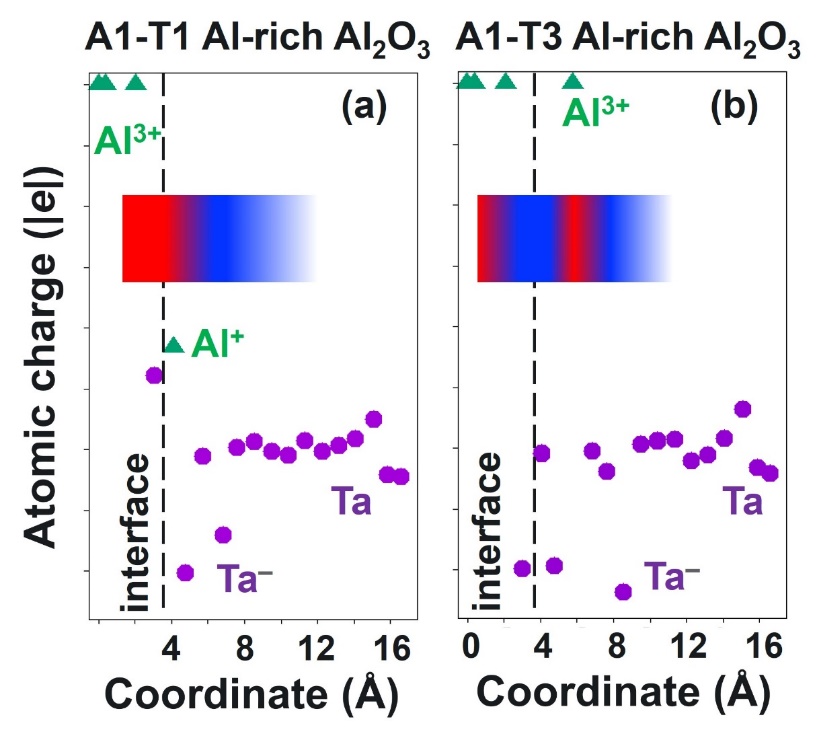


**Figure S7.** Charge distribution in selected Ta on Al-rich Al_2_O_3_ systems represented by layer-averaged Bader atomic charges of the Al and Ta species: intermixed configurations A1-T1 (a) and A1-T3 (b). The locations of Ta/Al_2_O_3_ interfaces are indicated with vertical dashed lines. The colored horizontal bars illustrate the variations of positive (red) and negative (blue) excess charge induced by the Al-Ta intermixing near the interface.


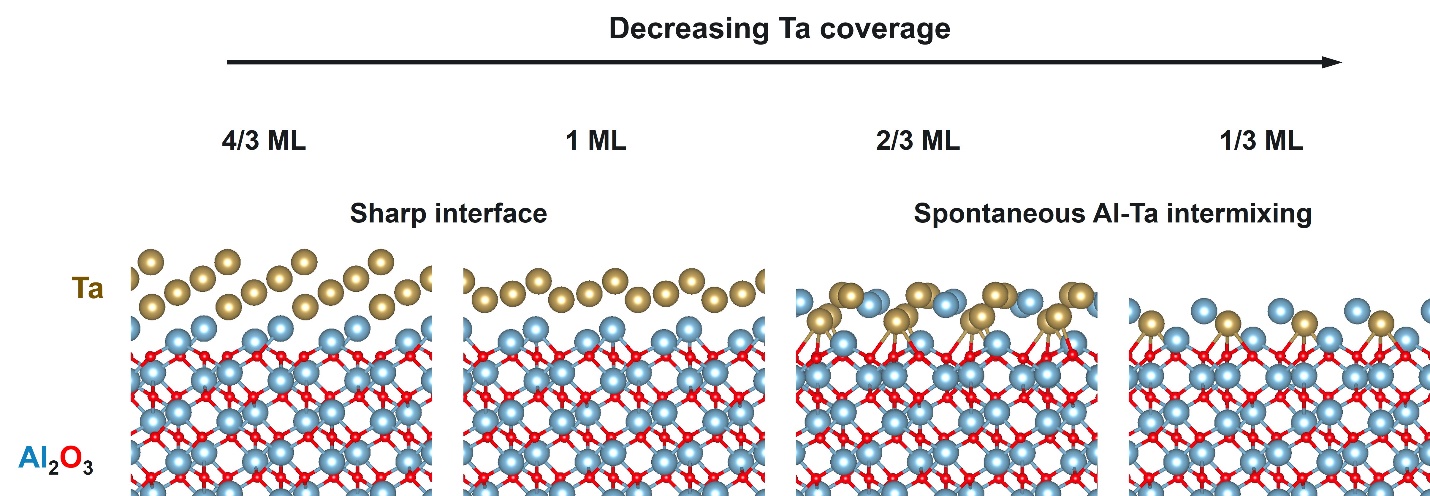


**Figure S8.** Layer-by-layer decreasing of the Ta coverage on the Al-rich surface causes a spontaneous Al-Ta rearrangement at the Ta coverage of ~2/3 ML. This observation indicates that in the reverse process, i.e., Ta deposition on the Al-rich Al_2_O_3_ (0001) surface, the surface Al and Ta atoms intermix at the early stages of Ta deposition resulting in the dissolution of the top al plane into the Ta film.


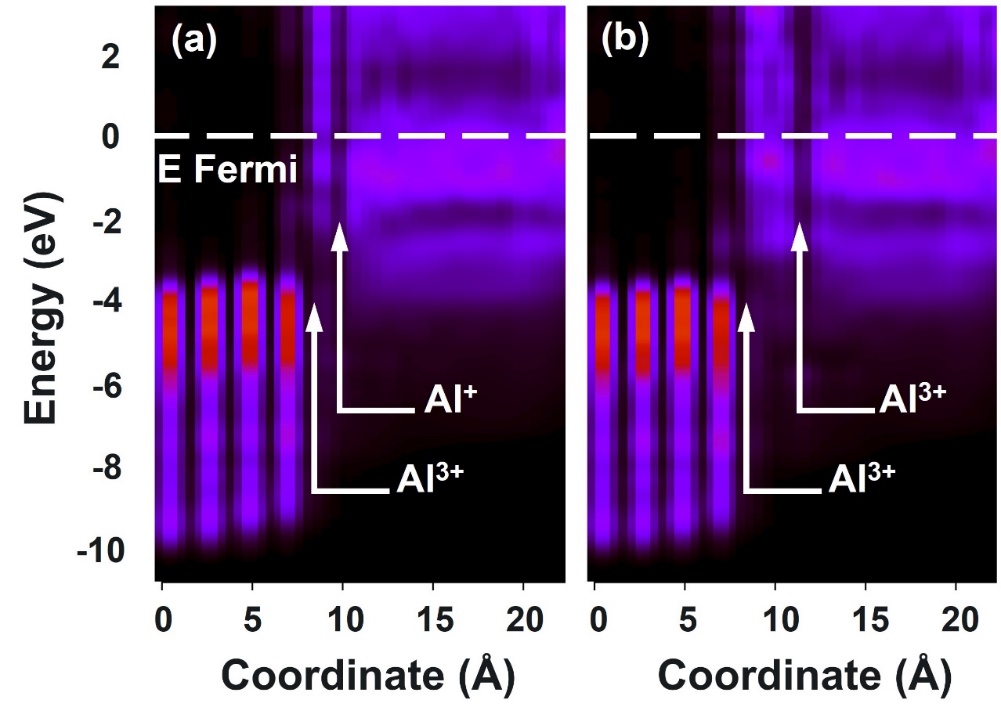


**Figure S9.** Heatmap representation of one-electron density of states calculated for selected Ta/Al_2_O_3_ systems and projected on the Al, O, and Ta atomic planes. Ta on Al-rich Al_2_O_3_: intermixed configurations A1-T1 (a) and A1-T3 (b), where A1 and T1 are the first Al and Ta planes near the interface, respectively, and T3 is the third Ta plane from the interface. All energy scales are aligned so that Fermi energy is at 0 eV. The substitutional Al impurities in Ta, arranged in planar configurations in (a) and (b), induce DOS depletion in the Ta film.

**Table S1. Comparison of findings on pure Ta (BCC structure) superconducting films.**

| **Substrate** | **Deposition method** | **Film orientation (out of plane direction)** | **Ta thickness (nm)** | **Substrate treatment & deposition conditions** | **Tc [K]** | **RRR value** |
| --- | --- | --- | --- | --- | --- | --- |
| Sapphire  (0001) ^[8]^ | DC magnetron sputtering | Ta (110) | 120 | Annealed up to 1100 °C (prior to deposition) | 4.2 | 4.5 |
| Sapphire  $(11\bar{2}0)$ ^[9]^ | RF magnetron sputtering | Ta (110) | 150 | Deposited at 750 °C | 4.18 | 7.5 |
| Sapphire  (0001) ^[10]^ | RF magnetron sputtering | Ta (222) | 120 | Deposited at 750 °C | 4.3 | 19.36 |
| Sapphire  $(11\bar{2}0)$ ^[10]^ | RF magnetron sputtering | Ta (110) | 120 | Deposited at 750 °C | 4.28 | 7.48 |
| Sapphire  $(11\bar{2}0)$ ^[11]^ | Molecular beam epitaxy | Ta (110) | 30 | 200 °C for 2 h and followed by 850 °C for 0.5 h to remove surface contamination (prior to deposition) and Ta  deposited at 550 °C | 4.12 | 9.53 |
| Silicon (100) ^[12]^ | DC magnetron sputtering | Ta (110) | Unknown | cleaned inside the growth chamber at 500 °C (prior to deposition), then TiN_x_ buffer layers and Ta deposited at 500 °C | 3.9 | 3.85 |
| Silicon (100) ^[13]^ | Sputtering | Ta (110) | 100 | Deposited at 400 °C | 2.9 | Unknown |
| Sapphire  (0001) ^[14]^ | DC magnetron sputtering | Ta (222) | 100 | Deposited at 630 °C | 4.35 | 47 |
| Sapphire  (0001) * | RF magnetron sputtering | Ta (222) | 30 | Deposited at 750 °C | 3.84 | 4.98 |

*This work

**References:**

1. Glavic, A.; Bjorck, M. GenX 3: the latest generation of an established tool. *J. Appl. Crystallogr.* **2022**, *55* (4), 1063-1071. doi:10.1107/S1600576722006653.
2. Kresse, G.; Furthmüller, J. Efficient iterative schemes for ab initio total-energy calculations using a plane-wave basis set. *Phys. Rev. B. 1996,* 54:11169–11186. [https://doi.org/10.1103/PhysRevB.54.11169](https://urldefense.com/v3/__https:/doi.org/10.1103/PhysRevB.54.11169__;!!P4SdNyxKAPE!BWgdTRxd5Lm1H7oB3WNpcp9m6VwSjPQrNoAaRH5_-ivFtGXYgahJT-RA4B9ABFgkn6AyOtrRQZzYO7IhNy9qaIti8yo$).
3. Kresse, G.; Joubert, D. From ultrasoft pseudopotentials to the projector augmented-wave method. *Phys. Rev. B.* 1999,59:1758–1775. [https://doi.org/10.1103/PhysRevB.59.1758](https://urldefense.com/v3/__https:/doi.org/10.1103/PhysRevB.59.1758__;!!P4SdNyxKAPE!BWgdTRxd5Lm1H7oB3WNpcp9m6VwSjPQrNoAaRH5_-ivFtGXYgahJT-RA4B9ABFgkn6AyOtrRQZzYO7IhNy9qYDeApds$)
4. Perdew, J.P.; Ruzsinszky, A.; Csonka, G.I.; Vydrov, O.A.; Scuseria, G.E.; Constantin, L.A.; Zhou, X.; Burke, K. Restoring the density-gradient expansion for exchange in solids and surfaces. *Phys. Rev. Lett.* 2008*,* 100:136406. [https://doi.org/10.1103/PhysRevLett.100.136406](https://urldefense.com/v3/__https:/doi.org/10.1103/PhysRevLett.100.136406__;!!P4SdNyxKAPE!BWgdTRxd5Lm1H7oB3WNpcp9m6VwSjPQrNoAaRH5_-ivFtGXYgahJT-RA4B9ABFgkn6AyOtrRQZzYO7IhNy9qXJkB3Fg$).
5. Blöchl, P.E. Projector augmented-wave method. *Phys. Rev. B.* 1994, 50:17953–17979. [https://doi.org/10.1103/PhysRevB.50.17953](https://urldefense.com/v3/__https:/doi.org/10.1103/PhysRevB.50.17953__;!!P4SdNyxKAPE!BWgdTRxd5Lm1H7oB3WNpcp9m6VwSjPQrNoAaRH5_-ivFtGXYgahJT-RA4B9ABFgkn6AyOtrRQZzYO7IhNy9qI7cW4Ws$)
6. Tang, W.; Sanville, E.; Henkelman, G. A grid-based Bader analysis algorithm without lattice bias. *J. Phys. Condens. Matter*. 2009, 21:084204.
7. Yu, M.; Trinkle, D.R. Accurate and efficient algorithm for Bader charge integration. J. Chem. Phys. 2011, 134:064111. [https://doi.org/10.1063/1.3553716](https://urldefense.com/v3/__https:/doi.org/10.1063/1.3553716__;!!P4SdNyxKAPE!BWgdTRxd5Lm1H7oB3WNpcp9m6VwSjPQrNoAaRH5_-ivFtGXYgahJT-RA4B9ABFgkn6AyOtrRQZzYO7IhNy9qxJ4Tegs$)
8. Wang, C.; Li, X.; Xu, H.; Li, Z.; Wang, J.; Yang, Z.; Mi, Z.; Liang, X.; Su, T.; Yang, C.; et al. Towards practical quantum computers: transmon qubit with a lifetime approaching 0.5 milliseconds. *npj Quantum Inf.* **2022**, *8* (1), 3. DOI: 10.1038/s41534-021-00510-2.
9. Zhou, C.; Mun, J.; Yao, J.; Anbalagan, A. k.; Hossain, M. D.; McLellan, R. A.; Li, R.; Kisslinger, K.; Li, G.; Tong, X.; et al. Ultrathin Magnesium-Based Coating as an Efficient Oxygen Barrier for Superconducting Circuit Materials. *Adv. Mater.* **2024**, *36* (18), 2310280. DOI: <https://doi.org/10.1002/adma.202310280>.
10. Yao, J.; Zhou, C.; Lozano, P. M.; Paone, S.; Liu, M.; Li, Q. Magnetic ac susceptibility of superconducting Ta films for quantum computing. *Supercond. Sci. Technol.* **2024**, *37* (1), 015010. DOI: 10.1088/1361-6668/ad10b6.
11. Jia, H.; Zhou, B.; Wang, T.; Wu, Y.; Yang, L.; Ding, Z.; Li, S.; Cai, X.; Xiong, K.; Feng, J. Investigation of the deposition of α-tantalum (110) films on a-plane sapphire substrate by molecular beam epitaxy for superconducting circuit. *J. Vac. Sci. Technol. B* **2023**, *41* (5). DOI: 10.1116/6.0002886.
12. Wu, Y.; Ding, Z.; Xiong, K.; Feng, J. High-quality superconducting α-Ta film sputtered on the heated silicon substrate. *Sci. Rep.* **2023**, *13* (1), 12810. DOI: 10.1038/s41598-023-39420-y.
13. Lozano, D. P.; Mongillo, M.; Piao, X.; Couet, S.; Wan, D.; Canvel, Y.; Vadiraj, A. M.; Ivanov, T.; Verjauw, J.; Acharya, R.; et al. Manufacturing high-Q superconducting α-tantalum resonators on silicon wafers. arXiv:2211.16437.
14. McFadden, A. P.; Oh, J.-S.; Zhou, L.; Larson, T.F.Q.; et al. Interface-sensitive microwave loss in superconducting tantalum films sputtered on c-plane sapphire. arXiv:2412.16730v1.
